# A pseudonymized corpus of occupational health narratives for clinical entity recognition in Spanish

**DOI:** 10.1186/s12911-024-02609-w

**Published:** 2024-07-24

**Authors:** Jocelyn Dunstan, Thomas Vakili, Luis Miranda, Fabián Villena, Claudio Aracena, Tamara Quiroga, Paulina Vera, Sebastián Viteri Valenzuela, Victor Rocco

**Affiliations:** 1 Department of Computer Sciences, Pontificia Universidad Catolica de Chile, Santiago, Chile; 2 Institute for Mathematical and Computational Engineering, Pontificia Universidad Catolica de Chile, Santiago, Chile; 3 Millennium Institute for Foundational Research on Data, Santiago, Chile; 4 Department of Computer and Systems Sciences, Stockholm University, Stockholm, Sweden; 5 Department of Computer Science, Universidad de Chile, Santiago, Chile; 6 Faculty of Physical and Mathematical Sciences, Universidad de Chile, Santiago, Chile; 7 Servicio de Salud del Maule, Ministerio de Salud, Talca, Chile; 8 Asociación Chilena de Seguridad, Santiago, Chile

**Keywords:** Natural language processing, Privacy, Named entity recognition, Corpus annotation

## Abstract

Despite the high creation cost, annotated corpora are indispensable for robust natural language processing systems. In the clinical field, in addition to annotating medical entities, corpus creators must also remove personally identifiable information (PII). This has become increasingly important in the era of large language models where unwanted memorization can occur. This paper presents a corpus annotated to anonymize personally identifiable information in 1,787 anamneses of work-related accidents and diseases in Spanish. Additionally, we applied a previously released model for Named Entity Recognition (NER) trained on referrals from primary care physicians to identify diseases, body parts, and medications in this work-related text. We analyzed the differences between the models and the gold standard curated by a physician in detail. Moreover, we compared the performance of the NER model on the original narratives, in narratives where personal information has been masked, and in texts where the personal data is replaced by another similar surrogate value (pseudonymization). Within this publication, we share the annotation guidelines and the annotated corpus.

## Introduction

The machine understanding of texts without a predefined structure (called free or unstructured text) is done using natural language processing (NLP). NLP is an area of artificial intelligence that deals with the interaction between humans and machines through language [1]. Clinical text is an important area of research in NLP, with a set of specific characteristics. For example, clinical texts see extensive use of non-standardized abbreviations, frequent misspellings due to the circumstances under which the data are produced, high variability across medical specialties and health professionals, and restricted availability for privacy reasons. Examples of clinical texts include anamneses, referrals, prescriptions, and notes of hospitalized patients [2]. Nowadays, it is used extensively in clinical informatics, and an example of that was its use during the COVID-19 outbreak [3, 4].

In 2017, NLP had a breakthrough with the appearance of transformers. In the hyper-cited paper by Vaswami et al. [5], the concept of self-attention was brought to the limit, replacing the recurrent networks that had been the dominant paradigm until then. The authors used a model with 165 million parameters, which easily surpassed the state of the art in a machine translation task. Since then, the NLP field has worked towards creating pre-trained language models (PLMs) that can be adapted for any specific downstream task. A prominent example is BERT (Bidirectional Encoder Representations from Transformers) [6]. BERT produces contextual word embeddings, which are numerical representations of words that depend on the context in which they are used. BERT is a masked language model trained by masking a word and predicting good word candidates to fill in the gap. This simple task, done on vast amounts of text, is known as pre-training. It allows the language model to acquire language understanding that can be leveraged for more specific downstream tasks [7].

As soon as the NLP field started to work in PLMs, clinical NLP introduced these tools into its set of techniques to improve performance in its tasks. Some examples of clinical PLMs are two different versions of ClinicalBERT [8, 9]. These models show a significant improvement in language tasks and a moderate improvement in prediction tasks. In Spanish, some publicly available PLMs are bsc-bio-ehr-es [10] and Clinical Flair [11]. These PLMs were primarily pre-trained with general and biomedical texts with some additions of clinical texts. The main reason behind this reliance on biomedical texts is that clinical texts are much more complex to obtain than biomedical publications. However, obtaining clinical texts is indeed possible. In a recent paper, our group fine-tuned bsc-bio-ehr-es using the most extensive clinical corpus reported for the Spanish language [12].

However, using models with many parameters can lead to undesired memorization of personally identifiable information (PII), such as personal identification numbers, names, or addresses. When memorization occurs, models become vulnerable to various attacks on privacy that leverage this phenomenon to extract memorized and often sensitive information. One category of attacks is known as membership attacks [13]. These attacks discern if a given datapoint was part of a model’s training data. Membership inference attacks can severely impact a person’s privacy when the dataset is highly sensitive, as is the case, e.g., for sexually transmitted diseases. Other forms of attack are data extraction attacks, in which the exact words in the dataset can be reconstructed [14], or record linkage attacks that aim to associate individuals with their sensitive attributes [15]. As we will discuss in depth in this article, anonymizing information requires much more than deleting columns with explicit personal identifiers, and the text cannot be left unscreened.

In this article, we work with anamneses of work-related accidents from the Chilean Safety Association (ACHS), which provides occupational health to 52% of Chilean workers. We believe it is important to use these types of texts as case studies because, in general, there are many machine-learning models in production in the health provider industry. In the era of ChatGPT, we find it essential to bring awareness to the prevalence and variability of sensitive information found in these types of documents and to share strategies for de-identifying them. In addition, our paper shows how a model trained to recognize clinical information in primary care data works when applied to occupational health data. Since language resources are generally scarce, the error analysis presented here can serve as a guide when designing text annotations.

This paper is organized as follows. First, we describe existing regulations for personal information in the US, Europe, and Chile. Then, we describe the basis of corpus annotation and existing resources. After that, our data and methods are described. A particular emphasis is placed on the annotation process, the pseudonymization, and the curation of the clinical annotations. Then, we present our results by comparing the performance of a named entity recognition model applied in three situations: to the original anamneses, to texts where PII has been masked, and when PII is replaced by other tokens (pseudonymization). Finally, we conclude with a discussion of our achievements, the limitations of our study, and suggestions for future work.

## Background

### Regulation for protecting personal health information

The Universal Declaration of Human Rights recognizes privacy as a fundamental human right, equilibrating the power an individual retains about themselves to the one possessed by governments and companies [16]. As the 2021 Annual Report of the United Nations High Commissioner mentions^1^: “The right to privacy is linked to the protection of human autonomy and personal identity”. Privacy protection is increasingly important in our current digital era, where data is often treated as a commodity, and even sensitive health information can be collected and sold by companies. For example, some apps process reproductive information and there are dating apps that ask users for their HIV status [17]. This information, if mishandled, can lead to discrimination and may severely impact human dignity.

In the United States, the Health Insurance Portability and Accountability Act (HIPAA)^2^, passed by Congress in 1996, establishes 18 classes of protected health information. The act stipulates that this protected information must be removed or masked for using electronic health records for research. The protected health information (PHI) classes are name, address, geographic location finer than the county, specific dates (e.g., birth or hospital admission), telephone number, email address, social security number, patient’s identification, bank account number, license number, medical device number, internet protocol (IP), username, passwords, biometric data, facial images and other characteristics that can uniquely identify a person. By setting comprehensive guidelines for healthcare providers, HIPAA provides a framework for how to work with PHI. A canonical example of the application of HIPAA to share healthcare data is MIMIC [18], a key contributor to advancing medical informatics in English.

In Europe, the General Data Protection Regulation (GDPR)^3^ has been applicable since 2018 and harmonizes data privacy laws across Europe. Among its recitals, we find: “The personal data should be adequate, relevant and limited to what is necessary for the purposes for which they are processed. This requires, in particular, ensuring that the period for which the personal data are stored is limited to a strict minimum. Personal data should be processed only if the purpose of the processing could not reasonably be fulfilled by other means”. The recitals also state that: “Every reasonable step should be taken to rectify or delete inaccurate personal data. Personal data should be processed to ensure appropriate security and confidentiality, including preventing unauthorized access to or use of personal data and the equipment used for the processing”. GDPR is considered the world’s strongest privacy and security law but fails to give clear instructions on manipulating PHI.

In Chile, the Patients’ Rights and Duties Law (No. 20,584) regulates individuals’ rights and duties concerning their healthcare actions, which came into force on October 1, 2012^4^. In particular, it establishes that every person shall have the right to make an informed choice to participate in scientific research. As the book Clinical Text Mining comments [2], this requirement is usually interpreted in various countries worldwide as meaning that if you cannot identify the person in the dataset, then you cannot contact that person to ask for consent to participate. Therefore, the data in such an anonymized state can be used for research, as in this study. In addition, Chile is currently drafting an update to the Personal Data Law (No. 19,628)^5^ establishing, among other things, that the data controller must adopt and prove that it has complied with all the quality and security measures necessary to ensure that the data is used exclusively for the purposes that were recollected. In addition, a Personal Data Protection Agency will be created, in the form of a public law corporation of a technical and decentralized nature. It should be noted that there is no robust definition of privacy in the current law, but different actors are influencing its drafting. As detailed in the next section, this study was heavily inspired by the HIPAA guidelines to annotate personally identifiable information.

### Corpus annotation for the automatic detection of clinical entities and personally identifiable information

A corpus (plural: corpora) is the Latin word for “body” and denotes a large sample of written or spoken language that is considered to be representative of either the standard or some geographic variant or some specific historical period [19]. This approximation can be expanded towards the computational field as a finite collection of machine-readable texts representing a language or a particular state of a language [20].

Many corpora contain word-level annotations that assign labels to words and phrases. For clinical language, common choices of tags are diseases, medications, or body parts. A Chilean example of such a corpus is the Chilean Waiting List Corpus^6^ which was released in 2022. This corpus consists of 9,000 primary care referrals annotated with ten entities, six attributes, and pairs of relations with clinical relevance [21, 22]. The Chilean Waiting List Corpus was used to train a model to automatically recognize clinical entities, with the results summarized in Baez et al. [23]. Part of the results presented in this publication were obtained by applying this model trained on primary care referrals to the occupational health corpus described in this paper.

Regarding privacy concerns, sharing clinical datasets is often difficult due to the sensitive nature of the data. Clinical datasets, particularly those containing unstructured free text, can contain many direct and indirect identifiers. These identifiers are examples of PII. The presence of PII means there is a risk that clinical information can be linked to an individual, making the sharing of such data dangerous from a privacy perspective. Direct identifiers, such as names and identification numbers, are directly associated with an individual. While these are typically the most sensitive category of PII, indirect identifiers can also be used to identify individuals. For example, knowing a patient’s age, their occupation, and where they live may be enough to single out a person.

To mitigate the privacy risks associated with PII in clinical data, these datasets are often *de-identified* before they are shared so that the information can no longer be used to identify any individual. This de-identification can be done manually by humans or automatically using machine learning algorithms. Systems for automatic de-identification typically rely on NER models that detect sensitive PII. Training effective NER models is only possible when there are training datasets of sufficient quality, relevance, and scale. When this is the case, privacy risks can be decreased without sacrificing the usefulness of the data both for fine-tuning and pre-training purposes [24, 25].

Creating high-quality NER datasets is a laborious process. Consequently, there is a lack of large datasets for PII detection in clinical data. This lack of datasets is especially dire in languages other than English. This study focuses on Spanish data, and very few clinical Spanish datasets are annotated for PII. MEDDOCAN [26] is a prominent example that is freely available, consisting of 1,000 fictional clinical narratives annotated with the realistic PII of non-existing patients. However, the data, particularly the distribution of PII, do not necessarily reflect the nature of real-world clinical data. The data presented in this study are not only annotated for clinical entities but are, as a consequence of the manual de-identification process, annotated for PII as well. These 1,787 documents are, therefore, of considerable value as they are not only based on real clinical data but are one of the largest PII datasets for clinical Spanish.

## Data and methods

**Fig. 1:**
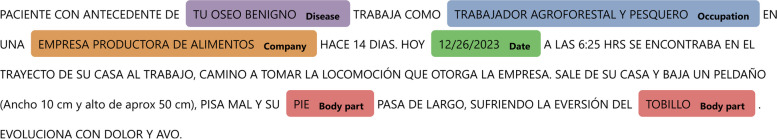
Example of an anamnesis from a work-related accident. Translation (with the abbreviations expanded in brackets): *Patient With a History of* *benign Bone Tu [tumor]* *works as an* *agroforest and fishery worker* *in a* *food producing company* *14 days ago. Today* *12/26/2023* *at 6:25 am He was on the trajectory from his house to work, on the way to take the locomotion provided by the Company. He Leaves His House and Goes Down a Step (Width 10 Cm and Height Approx. 50 Cm), Steps Badly and His* *foot* *Goes Along, Suffering the Eversion of the* *ankle. It Evolves With Pain and OVI [osteoarticular volume increase]*

This study presents a new dataset for clinical NER that covers both clinical entity recognition and detection of PII. This section describes where the data comes from and how it was annotated. Then, we describe the procedure for de-identifying the datasets so that they can be released. Figure 1 shows an example of what is achieved, a text from a work-related accident where PII has been replaced and clinical tags have been annotated. An English translation is provided in the caption.

### Anamneses from ACHS

The reports used in this project result from a collaboration between the Chilean Safety Association (ACHS) and the Institute for Foundational Research on Data (IMFD). ACHS receives patients who have suffered a labor-related accident or disease. When workers have an accident or suspect an occupational disease, they are admitted to one of their clinics. Upon admission, the admitting staff writes a report. After admission, a physician checks the patient and registers the relevant clinical information for mnemotechnical and legal reasons in a document called *anamnesis*. Every clinic’s medical and administrative board decides a final coverage rating for each accident or, in the case of diseases, if the disease is related to the applicants profession. The work presented here stems from the analysis of 1,787 documents registered between 2021 and 2022. Of these documents, 1,614 are anamneses from work-related accidents, and 173 are anamneses from work-related diseases.

### Annotation of clinical entities

For annotating clinical entities in the previously described corpus, we used a different strategy due to the limited availability of the medical doctor. We pre-annotated the entities *disease*, *medication*, and *body part* using a model previously published by our group [23, 27]. The physician then had to revise each text and modify the entities if she noticed a mistake.

In clinical texts, a frequent phenomenon is the presence of *nested entities*, where an entity can be found totally or partially contained in another entity [27]. For example, “colon cancer” is a disease that includes the body part “colon” as well as the disease “cancer”. Our group dealt with nested NER proposing a Multiple LSTM-CRF model that achieves the best results over the annotated Chilean Waiting List Corpus [21], which is a corpus made from medical and dental referrals written in primary care.

The work-related texts were pre-annotated using the aforementioned model. The physician (who has five years of experience) was tasked with modifying the annotations when she found mistakes. This included removing erroneous annotations and adding new ones that were missing.

### Annotating personally identifiable information

Two Ph.D. students (LM and TQ) from the Department of Computer Science annotated PII in 1,787 texts following an annotation guideline created for this project^7^. This guideline was inspired by the MEDDOCAN project [26], adapted to the Chilean context, and revised by a linguist. The annotations were performed using the software INCEpTION [28]. Our guidelines were used in a related work [29].

The annotated PII classes were last names, first names, occupations, company names, locations, dates, health care units, RUN numbers, ID numbers, phone numbers, emails, and ages. This set of PII classes was motivated by prior work [26, 30] and was adapted to the specific context in which our data were collected. RUN numbers were added as these are direct identifiers specific to Chile. Occupations and company names were considered since they are indirect identifiers that are frequent in the data.

For the curation of the corpus, a third Ph.D. student (CA) met with the two annotators and, side by side, analyzed the annotations. In case of disagreement, they decided on the correct assignment based on the guidelines. During the curation, the guidelines were slightly adjusted to better respond to the situations found during these discussions. The consolidation of the 1,787 texts took over 12 hours, and it was done in several days. It is important to note that we did not use automatic pre-annotation methods; each narrative was manually annotated from scratch.

### De-identification procedure

After manually annotating the ten different categories of PII, the corpus was sanitized using two de-identification methods. The first method replaced the sensitive entities by their class name. For example, a last name was replaced with the placeholder value [Last Name].

The corpus was also de-identified by replacing sensitive entities with *pseudonyms*^8^. These pseudonyms were selected to obscure the details of the PII while preserving higher-level semantics. The method for choosing pseudonyms varied depending on the type of PII. Names were replaced with other common Chilean names, while gender information was preserved in cases where the name’s gender could be guessed.

Occupations were mapped to the International Standard Classification of Occupations (ISCO) established by the United Nations ILOSTAT. This was done in an automated fashion by feeding every unique occupation to OpenAI’s Chat Completion API (running GPT 3.5) for few-shot classification. The prompt included a problem description and examples of correct classifications done manually. The classifications were also done manually when ChatGPT could not classify the occupation. Then, each occurrence of an occupation was replaced by a placeholder specific to the occupation category. For example, *mecánico industrial* (industrial mechanic) was mapped to *técnico* (technician). A fallback value of *trabajador* (worker) was used when no suitable replacement could be generated.

Company names were processed similarly to occupations but done entirely manually. A list of individual companies in the data was assembled. Each company was looked up manually by searching for the company name online. The companies were categorized according to their industry based on the information available online and the information inferred from the clinical data. Each company was then replaced with a placeholder value. For example, a school would be replaced with the value *un centro educativo* (a center for education). If no suitable replacement could be found, the fallback *una empresa* (a company) was used.

Healthcare units were also replaced by mapping each occurrence in the corpus to a category of healthcare institution. For example, a specific hospital would be mapped to the generic description *un hospital* (a hospital). This was also used as a fallback when the category could not be inferred.

Names and locations were replaced using word lists gathered for each category of name and location. For first names, an attempt was made to preserve the gender if it could be inferred. Names were randomly sampled from lists of Chile’s 100 most common first and last names. For locations, the pseudonymizer distinguished between five different location types that were manually chosen for each location in the corpus. Each location type was associated with a list of relevant locations in Chile from which a replacement was selected randomly. When the location class was unknown, the replacement *un lugar* (a place) was used.

Ages and dates were replaced by shifting the values. For ages, the value was shifted by a number of years randomly selected for each entity. Dates were changed to occur between years 2017 and 2023 and were shifted a random amount of days if it was possible to infer the full date. For weekdays, a random new weekday was selected.

RUN, ID, and phone numbers were also replaced. The replacements were selected randomly. For IDs, a random string of alphanumerical characters was generated while preserving the structure of the original ID. For RUN and phone numbers, a random valid replacement was generated.

## Results

The procedures described in the previous section resulted in a corpus annotated with clinical entities and PII. This section describes the findings from the annotation procedure and provides statistics that describe the corpora. Furthermore, the value of the corpus and the de-identification strategies is demonstrated through an experiment in which a model for clinical NER is evaluated on three versions of the corpus.

### Inter-annotator agreement for PII

The inter-annotator agreement was assessed using the pair-wise F_1_ score [31, 32] between both annotators and with the gold standard attained after curating the annotations from both annotators. The scores for each pair are listed in Table 1.

**Table 1:** Inter-annotator agreement for annotations of PII between the annotators (A1 & A2) and with the curated gold standard (GS). The values are pair-wise F_1_ scores, and the support is the number of instances of each PII

Entity class	Support	F_1_ score		
		GS - A1	GS - A2	A1 - A2
Global (micro avg)	5,460	0.92	0.92	0.86
Occupation	1960	0.90	0.92	0.83
Full Date	1105	0.98	0.97	0.95
Date Part	859	0.96	0.92	0.88
Health Care Unit	604	0.90	0.89	0.83
Company	317	0.85	0.84	0.74
Age	280	0.96	0.94	0.92
Last Name	136	0.93	0.92	0.86
Location	108	0.78	0.79	0.61
First Name	65	0.92	0.87	0.79
ID	13	0.96	0.69	0.67
Personal ID	8	0.88	0.93	0.80
Phone Number	3	1.00	0.80	0.80
Email	2	1.00	0.00	0.00

From the analysis of disagreement between annotators, we found that the *Occupation* label presents an additional layer of complexity, as certain words may refer to both actions and occupations depending on the context. For instance, “works in cleaning” suggests an occupation, whereas “was cleaning” implies an action. *Full Date* entities demonstrate consistently good results, likely owing to their easily identifiable and structured token format. *Date part* entities present a slightly higher ambiguity than full dates but still follow discernible patterns.

*Health Care Unit* entities are straightforward to identify, as they typically adhere to a clear structure involving descriptors like “Hospital/Clinic” followed by a location, like “Hospital La Cisterna”. Conversely, the tags *Institution* and *Location* labels pose challenges when the company name indicates the location where the action occurred.

Entities such as *Age* prove easy to identify, commonly appearing as expressions like “number + years” or explicitly labeled as “Age: number”. Similarly, *Last Name* entities are well identified, with a higher occurrence compared to *First Name*, aligning with expectations in contexts such as doctor mentions, where the convention is to refer to individuals by their last names (e.g., “Dr. Gonzalez”). Identification numbers did not appear often. When they did, we distinguished between *Personal ID* (which in Chile is called *Rol Único Nacional (RUN)* or Unique National Number) and the entity *ID* used to annotate license numbers or sheet numbers. Lastly, *Phone number* and *Email* entities exhibit low occurrence and possess highly structured text, facilitating the identification.

### Corpus statistics

Table 2 provides a detailed analysis of the statistics of the annotated corpus. It consists of 1,787 documents, where 1,614 are anamneses after an accident and 173 anamneses after the suspicion of an occupational disease. Notably, the data reveals a nearly twofold prevalence of medical entities compared to PII entities.

**Table 2:** Corpus statistics

Metric	Total
Documents	1,787
Tokens	221,854
Vocabulary	20,779
Lexical diversity	9.4$\%$
Tok. per doc.	124± 93
Ent. per doc.	8.6 ± 5.7
Annotated tokens	27,036
PII Entities	5,460
Medical Entities	10,019

Table 3 shows this corpus’s frequency of annotated entities. We observe how the clinical entities are far more common than any personally identifiable category.

**Table 3:** Frequency of occurrence of the PII categories and the clinical tags

Entity class	Frequency
Occupation	1960
Full Date	1105
Date Part	859
Health Care Unit	604
Company	317
Age	280
Last Name	136
Location	108
First Name	65
ID	13
Personal ID	8
Phone Number	3
Email	2
Body part	5,061
Disease	3,026
Medication	1,932

**Fig. 2:**
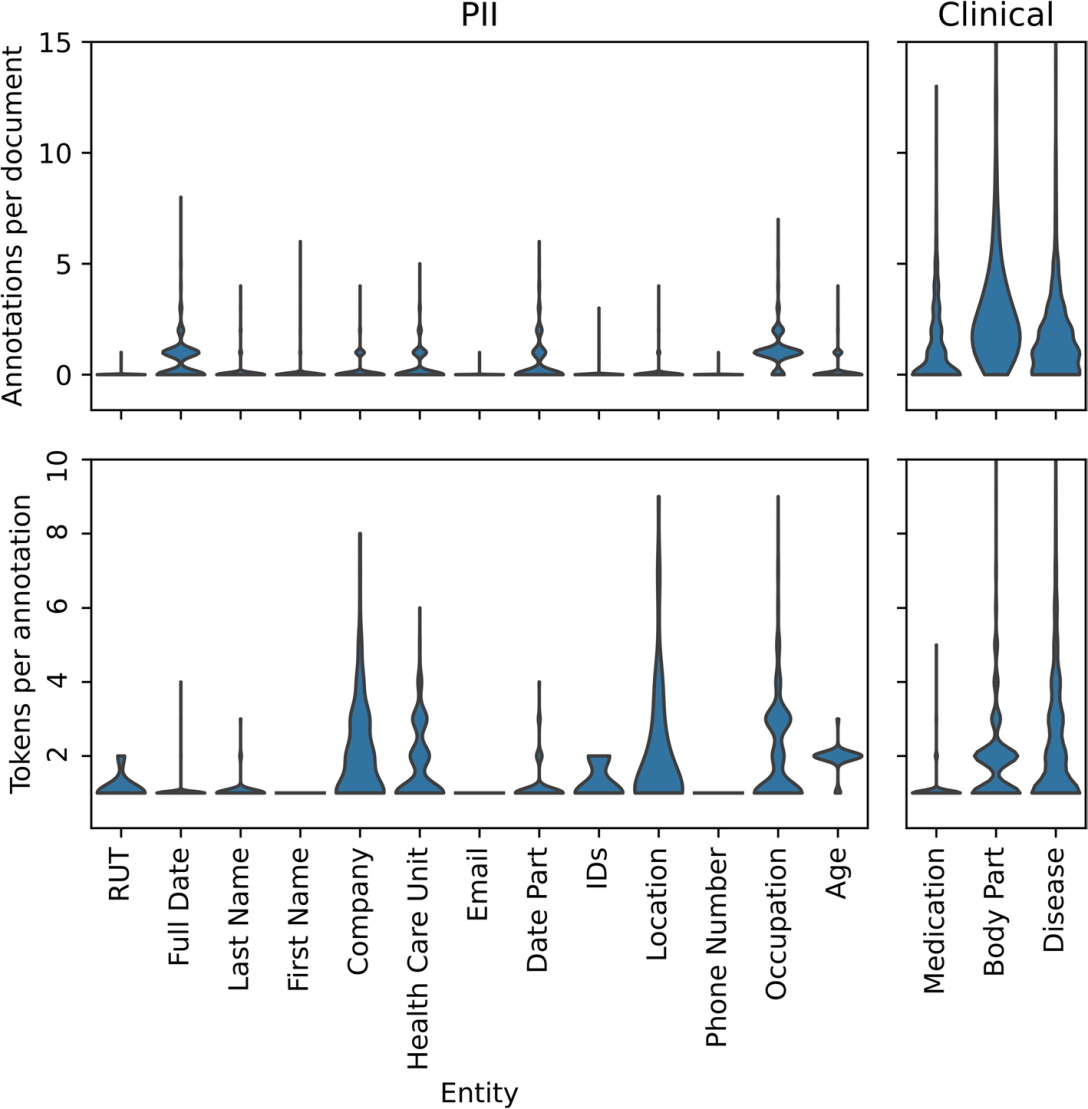
Frequency distribution of annotated entities per document (upper) by subcorpus, and tokens per entity across the subcorpus (lower). Some entities have an out-of-bounds maximum. Body part has a maximum of 30 annotations per document and 13 tokens per document, and Disease has a maximum of 17 annotations per document and 20 tokens per annotation

Regarding token frequency, in Fig. 2, we can see the predominance of clinical annotations per document and varying distributions for the PII. When looking at the tokens per annotations, we observe how entities are composed of a single token. Conversely, tags such as location, occupation, and body part tend to be more extended entities.

### Evaluations using the new clinical NER corpus

To demonstrate the value of our clinical annotations, the new corpus was used to evaluate the clinical NER model trained using the Chilean Waiting List corpus. The evaluation was run using three different versions of the corpus: one in which sensitive PII was masked with class labels, one in which the PII was replaced with pseudonyms, and one in which the PII was left unaltered.

**Table 4:** The utility of the dataset was tested by evaluating a clinical entity recognition system. The precision (P), recall (R), and F_1_ were calculated for every entity type using each version of the dataset

Entity	Original			Masked			Pseudonymized		
	P	R	F_1_	P	R	F_1_	P	R	F_1_
*Disease*	0.93	0.78	0.85	0.90	0.74	0.81	0.92	0.76	0.83
*Body part*	0.96	0.98	0.97	0.95	0.98	0.96	0.95	0.98	0.97
*Medication*	0.96	0.94	0.95	0.93	0.94	0.93	0.96	0.93	0.95

Table 4 shows the precision, recall, and F_1_ scores for the model. The values are calculated for each entity class and for all three versions of the corpus. Results are not very different between the three strategies, and further experiments are needed to make any definitive conclusions. A slightly worse performance was observed when evaluating the model using the masked version of the corpus. On the other hand, the evaluation using the pseudonymized corpus yields results that are very close to those that use the sensitive, unaltered data. This difference may be explained by the fact that the masked version introduces tokens that are unnatural and unseen by the model during training. In contrast, the pseudonymized version produces more natural-looking text that is more specific and closer to what the model was trained with.

## Conclusion

In this article, we share a unique resource: anamneses annotated with entities of clinical importance and where pseudonymization and masking preserve privacy. To detect personally identifiable information, two researchers localized sensitive tokens in each document, and the gold standard was constructed by curating each text with a third researcher. A medical doctor with five years of experience validated the clinical tags of diseases, medications, and body parts in the same documents. The corpus contains valuable annotations for two NER tasks: identification of clinical entities and detection of PII. This resource thus adds to the list of annotated corpora of occupational health, which are quite rare, especially in languages other than English [33].

In addition, we tested the change in performance of a named entity recognition (NER) model for clinical tags over three corpora: 1) the original anamneses; 2) whereupon finding a PII, the tokens were replaced by their class name; 3) and in a corpus where the PII is pseudonymized through replacement with realistic surrogate values. Our results show similar performance among the three strategies, with small deteriorations when using the de-identified corpora. Nevertheless, this impact on performance should be interpreted cautiously: as we performed a cross-corpus evaluation, the model was only trained once, and we cannot test whether differences are statistically significant. In any case, the measured differences are minimal. The utility of sharing the de-identified versions of the corpus greatly outweighs the small negative impact of sanitizing the PII. Furthermore, the masked version of the corpus could be re-pseudonymized using a more sophisticated pseudonymization algorithm, which may yield more natural and better-performing replacements.

Future work includes enlarging the number of annotated documents to train NER for PII detection with more examples. Moreover, we plan to add more layers of annotated entities over the same documents. In fact, we have secured funding to annotate mechanisms and forms of accidents and then code those using the terminology provided by the International Labour Organization. Coding massive quantities of accidents and detecting their origin is crucial to informing stakeholders and changing regulations. Particularly, we are interested in evaluating the gender perspective in accident prevention, the role of heat in them, and possible bias in machine learning models. In addition, we are starting to work on creating synthetic text to be prepared for possible stricter regulations.

Among the limitations of our work is that we need to add more anamneses from work-related diseases if we want to confidently de-identify those types of documents. The research presented moves us towards a robust algorithm that can mask or replace the PII in large amounts of clinical text. In the era of large language models, this type of pre-processing is a must if we want to advance the field while respecting patients’ privacy.

## Data Availability

Annotation guidelines are freely available at https://totoiii.github.io/clinical_deidentification_guideline/. The code used to pseudonymize the data is available at: https://github.com/touzen/achs-pseudonymization. The annotated corpus is available on request at: https://doi.org/10.5281/zenodo.10447555.

## Footnotes

1 https://www.ohchr.org/es/press-releases/2021/09/artificial-intelligence-risks-privacy-demand-urgent-action-bachelet

2 https://www.hhs.gov/hipaa/index.html

3 https://gdpr-info.eu/

4 https://www.supersalud.gob.cl/consultas/667/w3-propertyvalue-4185.html

5 https://derechocienciaytecnologia.uc.cl/2023/05/08/proyecto-de-ley-de-proteccion-de-datos-personales/

6 https://zenodo.org/record/7555181

7 Spanish version: https://totoiii.github.io/guia_deidentificacion_clinica/, and English translation: https://totoiii.github.io/clinical_deidentification_guideline/

8 The pseudonymizer is available at: https://github.com/touzen/achs-pseudonymization
